# Leptin receptor deficiency impedes metabolic surgery related-weight loss through inhibition of energy expenditure in db/db mice

**DOI:** 10.1186/s13098-024-01270-7

**Published:** 2024-02-01

**Authors:** Dan Tong, Jie Xiang, Wei Liu, Fang Sun, Lijuan Wang, Aidi Mou, Tingbing Cao, Qing Zhou, Mei You, Yingying Liao, Peng Gao, Daoyan Liu, Zongshi Lu, Zhiming Zhu

**Affiliations:** Department of Hypertension and Endocrinology, Center for Hypertension and Metabolic Diseases, Daping Hospital, Army Medical University, Chongqing Institute of Hypertension, Chongqing, China

**Keywords:** Roux-en-Y gastric bypass, Leptin receptor, Weight regain, AMPK

## Abstract

**Background:**

Roux-en-Y gastric bypass (RYGB) surgery is an effective metabolic surgery against diabetes and obesity. Clinical evidence indicates that patients with severe obesity have a poor curative effect in losing weight if they suffer from leptin or its receptor deficiency, but the underlying mechanism remains elusive. Here, we investigated the effect of leptin receptor deficiency on metabolic dysfunction in db/db mice treated by RYGB surgery.

**Methods:**

The db/db mice and their heterozygote control db/m mice were subjected to RYGB or sham surgery. Body weight, blood glucose, food intake and glucose tolerance were evaluated. Micro-PET/CT and histological analysis were performed to examine the glucose uptake of tissues and the fat changes in mice. The key factors in glucose and fatty acid metabolism were detected by western blot analysis.

**Results:**

Compared with the sham group, the db/db mice in the RYGB group showed more significant weight regain after surgical recovery and improvement in hyperinsulinemia and glucose tolerance. However, the total body fat and multiple organ lipid deposition of RYGB-treated db/db mice was increased. The underlying mechanism studies suggested that the activation of AMPK regulated GLUT4 to increase glucose uptake, but AMPK could not promote fatty acid oxidation through the JAK2/STAT3 pathway under leptin receptor deficiency in db/db mice.

**Conclusion:**

We conclude that leptin receptor deficiency impedes the AMPK activation-mediated fat catabolism but does not affect AMPK-related glucose utilization after metabolic surgery in db/db mice. This result helps select surgical indications for patients with obesity and diabetes.

**Supplementary Information:**

The online version contains supplementary material available at 10.1186/s13098-024-01270-7.

## Introduction

Obesity and type 2 diabetes (T2DM) have become the largest global public health burden [1]. Currently, there are 500 million people with diabetes and nearly 3 billion obese and overweight people in the world [2, 3]. Obesity frequently co-exists with T2DM, leading to the so-called “diabesity epidemic”. In general, obesity is a major risk factor for the development of T2DM [4, 5]. Obesity causes hyperinsulinemia and insulin resistance, which in turn leads to impaired glucose tolerance and target organ damage [6]. Therefore, weight loss can effectively prevent glucose dysmetabolism and even reverse the development of diabetes [7]. However, the causality between obesity and type 2 diabetes is still debated because a subset of metabolically healthy obese people never develop type 2 diabetes [8]. Similarly, increased adipose tissue lipogenesis improved glucose tolerance despite being obese and elevated blood fatty acids [9].

Currently, metabolic surgery is the most effective treatment for obesity and diabetes [10, 11]. The underlying mechanisms of metabolic surgery are involved in calorie restriction, improvement of insulin sensitivity, changes in gastrointestinal hormones, and regulation of intestinal flora and bile acids [12–14]. Interestingly, numerous studies demonstrated that the hypoglycemic effects of metabolic surgery are weight-independent [14–16]. Moreover, we have confirmed that metabolic surgery mediated activation of the AMPK pathway plays a crucial role in maintaining glucose homeostasis in an insulin-dependent manner [12]. As a key regulator of metabolic homeostasis, AMPK can promote the cell membrane translocation of glucose transporter 4 (GLUT4) to enhance tissue glucose uptake, as well as upregulate fatty acid oxidation (FAO) to promote energy expenditure [17]. Previous studies have shown that the metabolic benefits of AMPK activation partly depend on the leptin receptor; however, the underlying mechanism remains elusive [18]. Several studies demonstrated that leptin or its receptor deficiency causes rare, autosomal recessive forms of severe, early-onset obesity, furthermore, obese patients with leptin receptor mutation have poor weight loss or significant weight regain after metabolic surgery [19–22]. In this study, we explored the effect of metabolic surgery on glucose homeostasis and energy expenditure in leptin receptor deficiency diabetic obese mice (db/db) and elucidated the role of leptin receptors in hypoglycemic and weight loss.

## Materials and methods

### Animal

The male db/db mice (leptin receptor deficiency) and their heterozygote control db/m mice were obtained from Gempharmatech. All mice were housed under a controlled temperature (21–23 °C) with relative humidity (50–60%) in a 12/12-hour light-dark cycle. They were free to access to tap water and food. All experimental procedures were performed in adherence to the National Research Council’s Guide for the Care and Use of Laboratory Animals and the institutional animal care and research advisory committee at Daping Hospital, Army Medical University.

### Surgery

At 6 weeks of age, mice were randomly allocated to RYGB or sham surgeries. Mice were fasted for more than 12 h before surgery and were anesthetized using an isoflurane gas anesthesia machine (VME animal anesthesia machine, Matrx Company, USA). The skin was disinfected with 0.5% iodophor and the surgery was performed under sterile conditions. An appropriate incision was made in the middle of the upper abdomen, cut through the skin and muscle layers by layer to enter the abdominal cavity, the lower esophageal segment was disconnected from the gastric junction, the gastric stump was continuously sutured and sealed, the jejunum was cut off at 4–6 cm from the flexor ligament, and end-to-end anastomosis was performed between the esophageal end and the distal jejunum. A jejuno-jejunostomy was performed at a distance of 4–6 cm from the gastrointestinal anastomosis site. The sham surgery group mice underwent in situ anastomosis surgery after being severed at the above-mentioned anastomosis sites [23]. Mice were fasted for 72 h after surgery, and care was taken to keep them warm during this period. The success rate of surgery is around 80%.

### Physiological parameters

Body weight, food intake, and water intake were weekly measured before surgery and after surgery. The tail-cuff blood pressure was measured at the end of the experiment (Softron-98).

### Micro-CT analysis

To further investigate the effect of RYGB on fat in db/db mice, the fat volume and fat percentage were detected using micro-CT. Firstly, the mice were anesthetized using isoflurane. Through high-resolution imaging methods, CT-Scan can capture CT imaging from multiple angles, reconstruct three-dimensional surfaces, and finally evaluate fat using contrast characteristics [24]. All data were analyzed using the Avatar3 software (PINGSENG Healthcare).

### PET-CT analysis

The uptake and distribution of glucose in vivo were determined by 2-deoxy- 2-[18 F] fluoro-D-glucose ([18 F] FDG), and high-resolution PET/CT imaging. In brief, animals were anesthetized with isoflurane and injected with a certain amount of [18 F] FDG through the tail vein (mice, approximately 200 µCi). Following a 50-minute absorption period, the animals were anesthetized with isoflurane again and imaged on a small-animal integrated micro-PET/CT scanner (Super Nova ®PET/CT, PINGSENG Health Care). The CT was acquired over 360 projections using 70 kV and 600 µA on a CMOS detector and reconstructed using a modified algorithm. The PET image was acquired for 1 bed position (10 min) and reconstructed using ordered subset expectation maximization. Regions of interest were manually drawn for standard uptake value calculations. All data were analyzed using the Avatar3 software (PINGSENG Health Care).

### Tolerance test

Before all tests, animals were fasted overnight (20:00–8:00). The animals were given the oral glucose solution (1.5 g/kg for db/m mice, 1 g/kg for db/db mice) for OGTT, the intraperitoneal glucose solution (1.5 g/kg for db/m mice, 1 g/kg for db/db mice) for IPGTT, and the intraperitoneal insulin solution (0.75 U/kg) for IPITT. Blood glucose was measured using the One Touch Ultra (LifeScan) through tail-vein bleeding before and after the test. All tests were conducted on different days.

### Respiratory exchange ratio (RER) and energy expenditure (EE)

Firstly, the mice were weighed, and then placed separately in a monitoring cage at room temperature of 22 °C and fed for 24 h in a 12/12-hour light-dark cycle. The entire monitoring process was performed in the Comprehensive Laboratory Animal Monitoring System (CLAMS; Columbus Instruments, OH, USA). All data were analyzed using the Oxymax software package (Columbus Instruments). The RER and EE were calculated as follows:

RER = VCO2 / VO2; EE = (3.815 + 1.232 × RER) × VO2 × 0.001 (kcal/ [kg × h])

Where carbon dioxide output (VCO2) and oxygen uptake (VO2) were counted between each [12].

### Histological analysis

For Oil-red O staining, liver tissues were embedded in optimal cutting temperature compound before sectioning at -20 ˚C and cut into 10-µm thick sections. The sections were stained using a Modified Oil Red O Stain Kit (G1261, Solarbio). Adipose tissues from mice in each group were fixed in 4% paraformaldehyde for 24 h at 4 °C and then embedded in paraffin. The 5 μm thick sections of adipose tissues were obtained and then stained with hematoxylin-eosin (HE). Images were observed using a Nikon E200 light microscope.

### Serum biochemical measurements

The mice were sacrificed, and then blood samples were collected to obtain serum. The levels of insulin and adiponectin in serum were detected using ELISA kits (Mercodia, 10-1247-01; Solarbio). The serum levels of total cholesterol (TC), triglycerides (TG), high-density lipoprotein cholesterol (HDL-C), and low-density lipoprotein cholesterol (LDL-C) were measured using the biochemical detection method (Mindray, BS-360 S).

### Western blot

The frozen tissues were successively pulverized and lysed in RIPA buffer (65 mM Tris-HCl, 150 mM NaCl, 1% NP-40, 0.5% sodium deoxycholate, and 0.1% SDS, pH 7.5), protease inhibitor cocktail tablets (04693132001, Roche) and phosphatase inhibitor tablets (4,906,837,001, Roche). After centrifugation, the supernatants were transferred into fresh tubes and protein concentrations were determined using the BCA method. Proteins (20 µg per lane) were separated on 10% SDS–PAGE gels and then transferred to PVDF membranes (IPVH00010, Millipore). After blocking, the filters were incubated with the following primary antibodies: GCK (PA5-92873, Invitrogen), PFKL (8175, Cell Signaling Technology), PKLR (ab171744, Abcam), ACLY (MA5-17027, Invitrogen), FASN (ab22759, Abcam), SREBF1 (14088-1-AP, Proteintech), CPT1A (DF12004, Affinity), AMPK (MA5-15815, Invitrogen), Phospho-AMPK (PA5-36615 Invitrogen), AKT (9272, Cell Signaling Technology), Phospho-AKT (9271, Cell Signaling Technology), STAT3(MA1-13042, Invitrogen), Phospho-STAT3(9145, Cell Signaling Technology), GLUT4(ab313775, Abcam) and β-actin (66009-1-Ig, Proteintech). After being washed and incubated with the appropriate horse radish peroxidase-conjugated secondary antibody (Santa Cruz Biotechnology), the immune complexes were visualized using a chemiluminescence reagent. Western blot results were densitometrically quantified with Quantity One software (Bio-Rad), and the intensity values were normalized to β-actin.

### Statistical analysis

Quantitative results are expressed as the mean ± SEM. All analyses were performed and graphs were created using GraphPad Prism 9. The differences between the two groups were analyzed using a two-tailed Student’s t-test. The differences among three or more groups were analyzed using one-way ANOVA followed by Bonferroni adjustment for multiple comparisons. Differences were considered significant when *P* values were less than 0.05. All images shown without biological replicates are representative of at least three independent experiments.

## Results

### RYGB decreased hyperglycemia but failed to reduce body weight in db/db mice

**Fig. 1:**
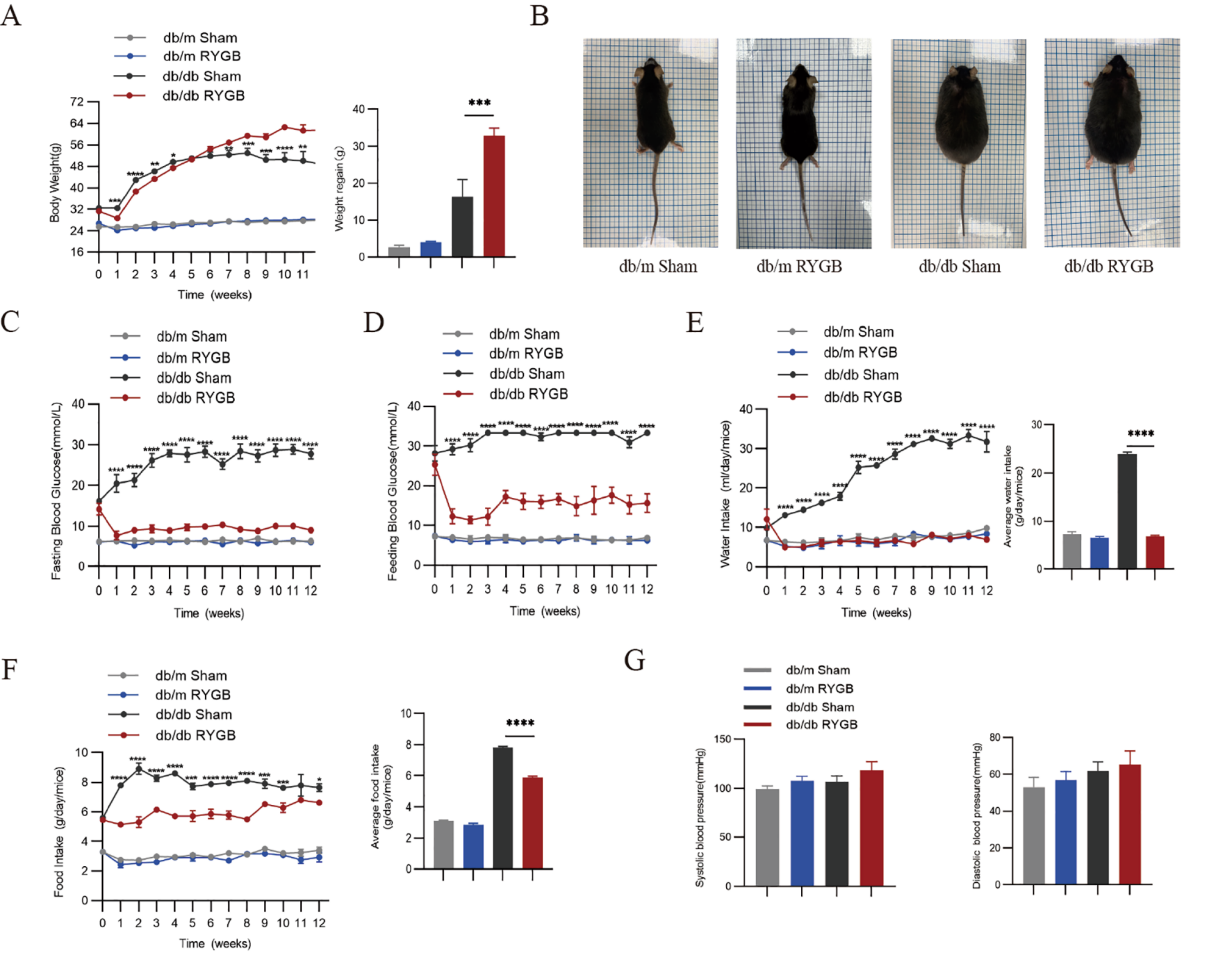
RYGB changes physiological parameters in db/m and db/db mice. **A**, Body weight. db/m and db/db mice were weighed after sham and RYGB surgery (*n* = 4–6). **B**, Whole appearance. **C-F**, Changes in food intake, water intake, fasting blood glucose and feeding blood glucose were monitored in db/m and db/db mice after sham and RYGB surgery weekly (*n* = 4–6). Daily average water intake and daily average food intake are shown on their right. **G**, Tail-cuff systolic blood pressure and diastolic blood pressure in db/m and db/db mice after sham and RYGB surgery (*n* = 5). The data are presented as the mean ± SEM. *p < 0.05, **p < 0.01, ***p < 0.001, ****p < 0.0001

Compared with db/m mice and the sham group, db/db mice showed a significant weight regain in db/db mice treated by RYGB during 12 weeks of follow-up surgeries (Fig. 1A, B). Importantly, RYGB significantly reduced hyperglycemia in db/db mice during both fasting and feeding status (Fig. 1C, D). After RYGB, average food and water intake were significantly decreased in db/db mice compared with the sham group (Fig. 1E, F). RYGB had no effects on blood pressure in mice (Fig. 1G). These collective results support that the hypoglycemic effect of metabolic surgery has less relationship with weight loss in db/db mice.

### Effect of RYGB on islet function in db/db mice

**Fig. 2:**
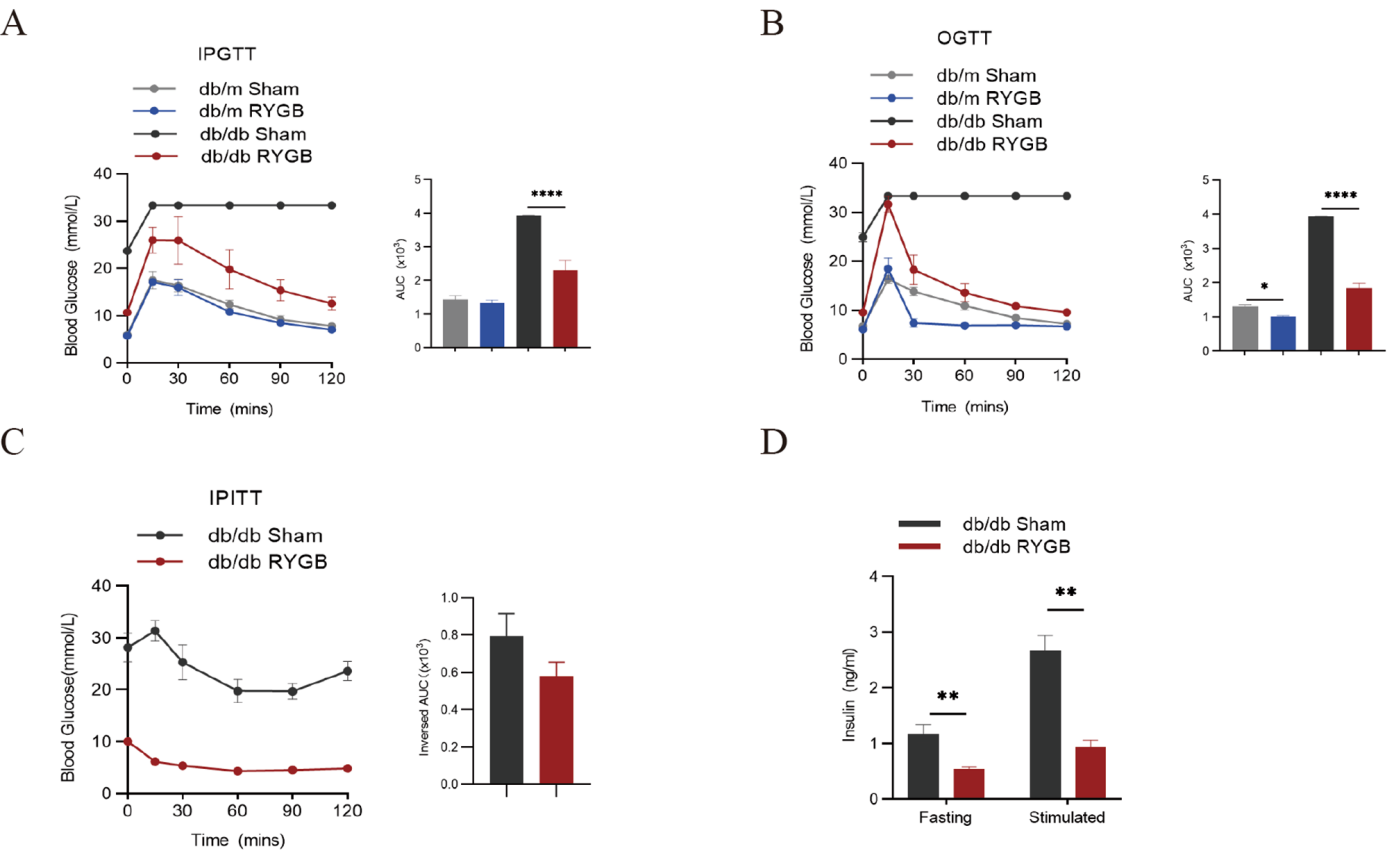
Effect of RYGB on islet function in db/db mice. **A** and **B**, Intraperitoneal glucose tolerance and oral glucose tolerance in db/m and db/db mice after RYGB and sham surgery (*n* = 4–5). The areas under the curve of each group are shown on their right. **C**, Insulin tolerance test in db/db mice after sham and RYGB surgery (*n* = 4). The inversed areas under the curve of each group are shown on their right. **D**, The fasting and stimulated insulin in sham- and RYGB-treated db/db mice (*n* = 4–6). The data are presented as the mean ± SEM. *p < 0.05, **p < 0.01, ****p < 0.0001

To further investigate the effect of RYGB on islet function, we examined the changes of blood glucose upon glucose loading and insulin challenge. There were abnormal fasting blood glucose and insulin levels, and intraperitoneal glucose tolerance test (IPGTT) as well as oral glucose tolerance test (OGTT) in db/db mice compared with db/m mice (Fig. 2A, B). However, RYGB significantly improved both IPGTT and OGTT in db/db mice compared with sham (Fig. 2A, B). Additionally, insulin sensitivity showed no difference during an insulin tolerance test (ITT) between sham and RYGB-treated db/db mice (Fig. 2C). Interestingly, RYGB markedly reduced plasma insulin levels in db/db mice (Fig. 2D). These results indicate that RYGB can markedly improve glucose homeostasis which is independent of plasma insulin levels.

### Effect of RYGB on fat distribution in db/db mice after RYGB

**Fig. 3:**
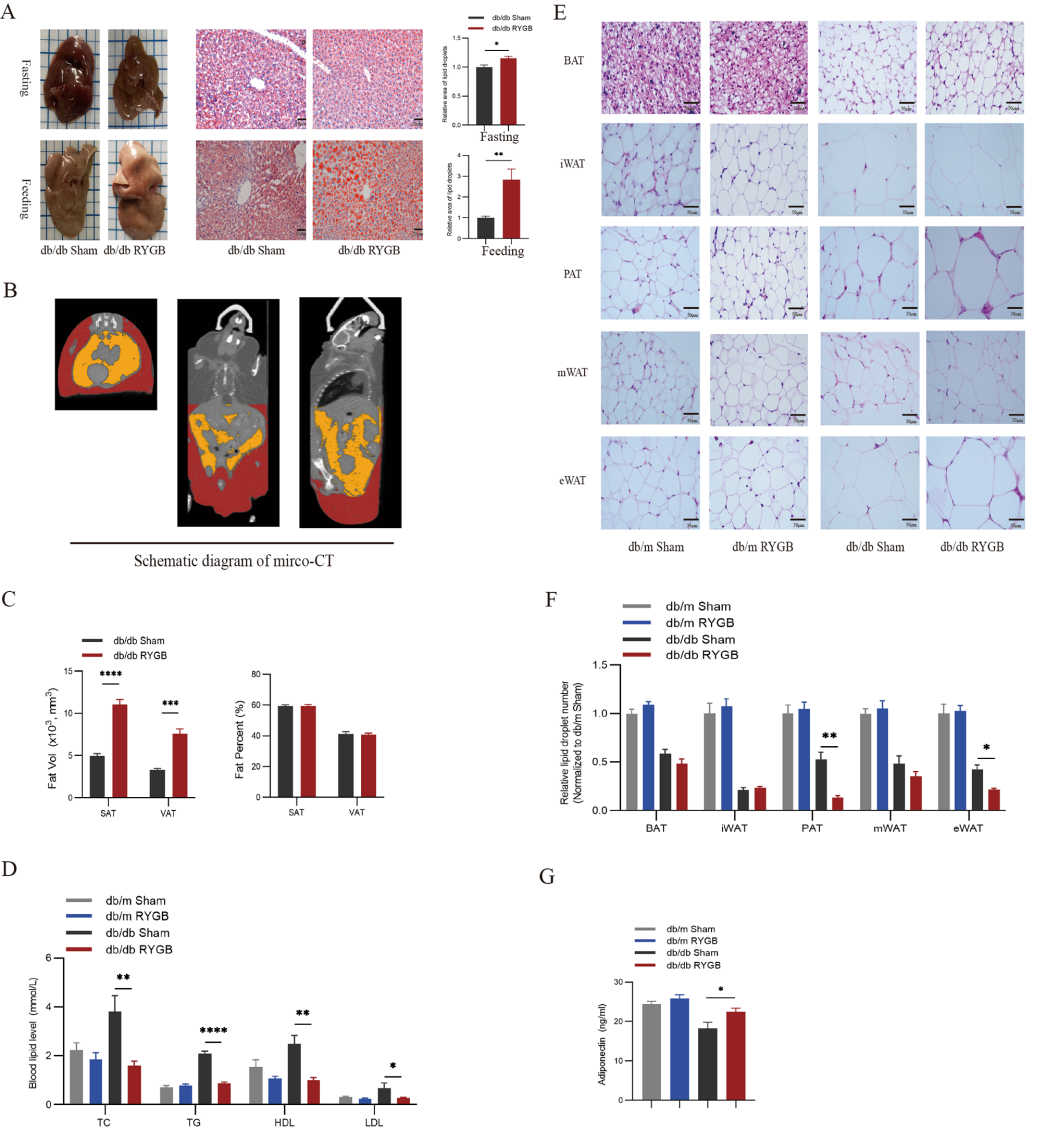
Effect of RYGB on fat distribution in db/db mice after RYGB. **A**, Representative images of fasting and feeding liver morphology and Oil red staining in db/db mice after sham and RYGB surgery. Scale bar, 50 μm. The quantitative results of the relative area of lipid droplets are shown on their right (*n* = 5). **B**, schematic diagram of micro-CT in RYGB-treated db/db mice. **C**, The subcutaneous and visceral fat volume and fat percent in db/db mice after sham and RYGB surgery (*n* = 4). **D**, Blood lipid levels in db/m mice and db/db mice after sham and RYGB surgery (*n* = 4). **E** and **F**, Representative images of H&E staining and quantitative results of lipid droplet number of BAT, iWAT, PAT, mWAT, and eWAT of db/m mice and db/db mice after sham and RYGB surgery (*n* = 5). Scale bar, 50 μm. **G**, The blood adiponectin in db/m mice and db/db mice after sham and RYGB surgery (*n* = 8). The data are presented as the mean ± SEM. *p < 0.05, **p<0.01,***p < 0.001, ****p < 0.0001. SAT, subcutaneous adipose tissue; VAT, visceral adipose tissue; TC, total cholesterol; TG, triglycerides; HDL-C, high-density lipoprotein cholesterol; LDL-C, low-density lipoprotein cholesterol; BAT, brown adipose tissue; iWAT, inguinal white adipose tissue; PAT, perirenal adipose tissue; mWAT, mesenteric white adipose tissue; eWAT, epididymis white adipose tissue

We further examined the fat distribution in db/db mice after RYGB. Unexpectedly, a more dominant fatty liver was found in db/db mice after RYGB. Liver oil red staining further showed that the intracellular lipid droplets were larger and had a higher lipid content than those in the sham group (Fig. 3A). To further determine the changes in fat volume, micro-CT was used to analyze systemic visceral and subcutaneous fat (Fig. 3B). Compared with the sham group, the volume of visceral and subcutaneous fat significantly increased in RYGB-treated db/db mice, but there was no difference in the percentage of total body fat (Fig. 3C). In contrast, the blood lipid levels of db/db mice were reduced after surgery (Fig. 3D). To confirm the obesity-accelerating effect of RYGB in db/db mice, we performed hematoxylin and eosin staining on brown adipose tissue (BAT), inguinal white adipose tissue (iWAT), perirenal adipose tissue (PAT), mesenteric white adipose tissue (mWAT) and epididymis white adipose tissue (eWAT) (Fig. 3E). The increase in fat mass was associated with larger adipocytes in RYGB db/db mice. There was no difference between the sham group and the RYGB group in BAT. In contrast, the number of lipid droplets in PAT and eWAT was significantly reduced in db/db mice with RYGB surgeries, compared to mice with sham surgeries (Fig. 3F). Adiponectin secreted by adipose tissue is closely related to fatty acid oxidation and blood glucose regulation. The adiponectin levels of RYGB-treated db/db mice were similar to those of db/m mice (Fig. 3G).

### RYGB increased glucose metabolism but did not affect fatty acid oxidation

**Fig. 4:**
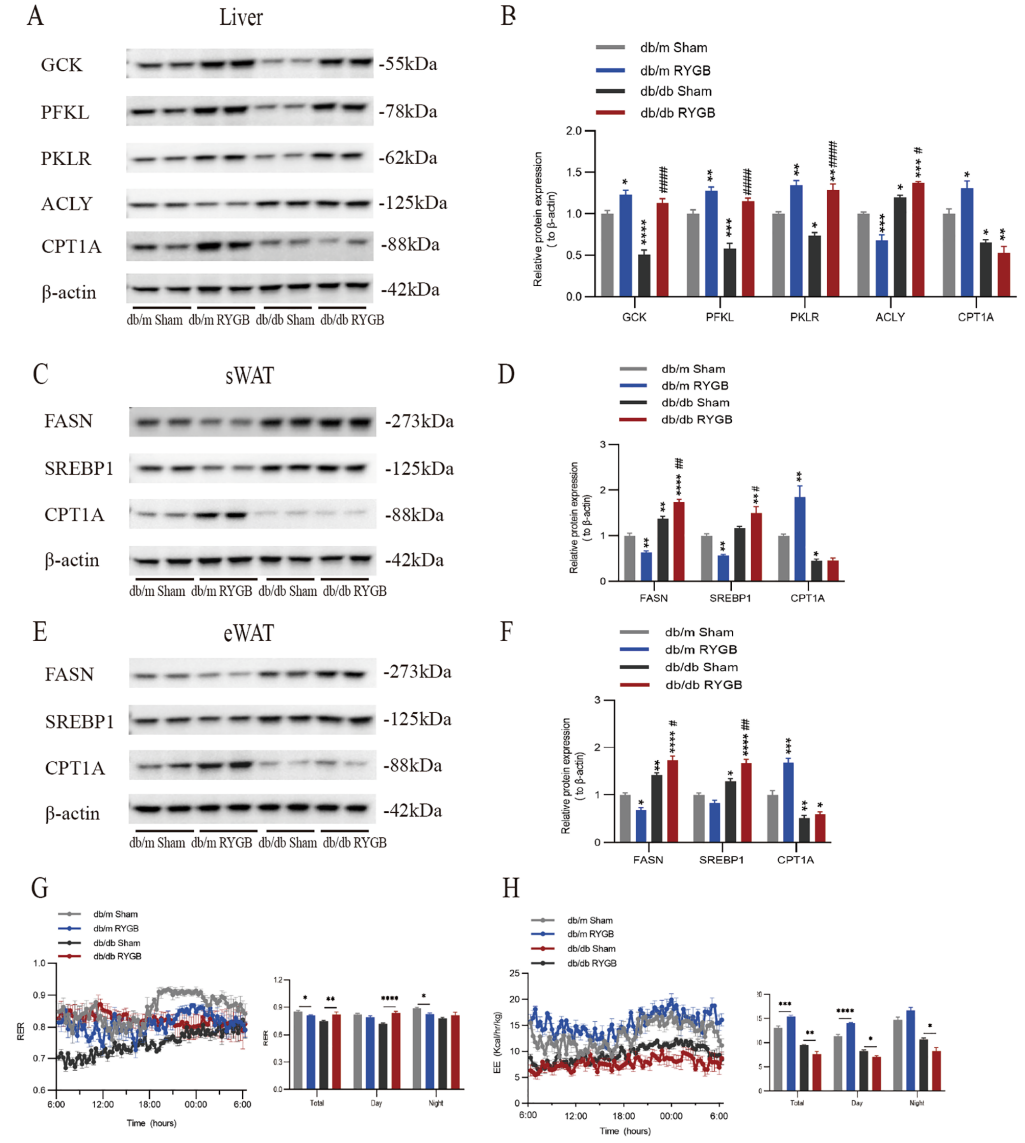
The changes of glucose and lipid metabolism in the liver and white adipose. **A** and **B**, Representative western blots and quantitative results of GCK, PFKL, PKLR, ACLY and CPT1A in the liver from db/m mice and db/db mice after sham and RYGB surgery (*n* = 4). Protein levels were normalized to those of β-actin. **C** and **D**, Representative western blots and quantitative results of FASN, SREBP1 and CPT1A in the sWAT from db/m mice and db/db mice after sham and RYGB surgery (*n* = 4). Protein levels were normalized to those of β-actin. sWAT, subcutaneous white adipose tissue. **E** and **F**, Representative western blots and quantitative results of FASN, SREBP1 and CPT1A in the eWAT from db/m mice and db/db mice after sham and RYGB surgery (*n* = 4). Protein levels were normalized to those of β-actin. eWAT, epididymal white adipose tissue. **G** and **H**, RER and EE values of db/m and db/db mice after sham and RYGB surgery (*n* = 4–6). Quantitative results are shown on the right. The data are presented as the mean ± SEM. *P < 0.05, **P < 0.01, ***P < 0.001, ****P < 0.0001, compared with the db/m sham group; #*P* < 0.05, ##*P* < 0.01, ###*P* < 0.001, ####*P* < 0.001, compared with the db/db sham group, by one-way ANOVA

To further investigate the effects of RYGB on glucose and fatty acid metabolism, we detected the changes in key metabolic enzymes in the liver and adipose tissue at the protein level. Compared to the sham group, RYGB increased the expression of glycolysis enzymes in the liver of db/m and db/db mice, including glucokinase (GCK), phosphofructokinase liver type (PFKL), and pyruvate kinase isozymes R/L (PKLR) (Fig. 4A, B). Importantly, the ATP-citrate lyase (ACLY) associated with fatty acid biosynthesis in the liver was significantly downregulated and the key enzyme carnitine palmitoyltransferase-1 (CPT-1) in fatty acid oxidation was significantly upregulated after RYGB in db/m mice (Fig. 4A, B). However, ALCY was significantly upregulated while CPT-1 didn’t change in leptin receptor deficiency db/db mice after RYGB (Fig. 4A, B). In subcutaneous white adipose tissue and epididymis white adipose tissue, enzymes involved in fatty acid synthesis, such as fatty acid synthesis (FASN) and sterol regulatory element-binding protein 1 (SREBP1), were decreased after RYGB in db/m mice, and CPT-1 was also upregulated (Fig. 4C-F). In contrast, FASN and SREBP1 were enhanced after RYGB in db/db mice, but CPT-1 was unchanged (Fig. 4C-F). To investigate the metabolic changes of RYGB in db/db mice, CLAMS was used to monitor the respiratory exchange ratio (RER) and energy expenditure (EE). The RER of RYGB-treated db/db mice was significantly increased, especially during the day, which may indicate that the proportion of glucose metabolism significantly increased after RYGB (Fig. 4G). Moreover, the EE of RYGB-treated db/db mice was significantly reduced (Fig. 4H), which indicated that the upregulation of the glucose utilization ratio could not induce an increase in total energy expenditure under leptin receptor deficiency. However, db/m mice with normal leptin receptors showed the opposite trend after RYGB (Fig. 4G-H). These results suggest that the surgical group is more easily weight regained than the sham in db/db mice, and the metabolic effect of RYGB in mice partly depends on the leptin receptor.

### Effect of RYGB on the AMPK-related JAK2-STAT3 pathway

**Fig. 5:**
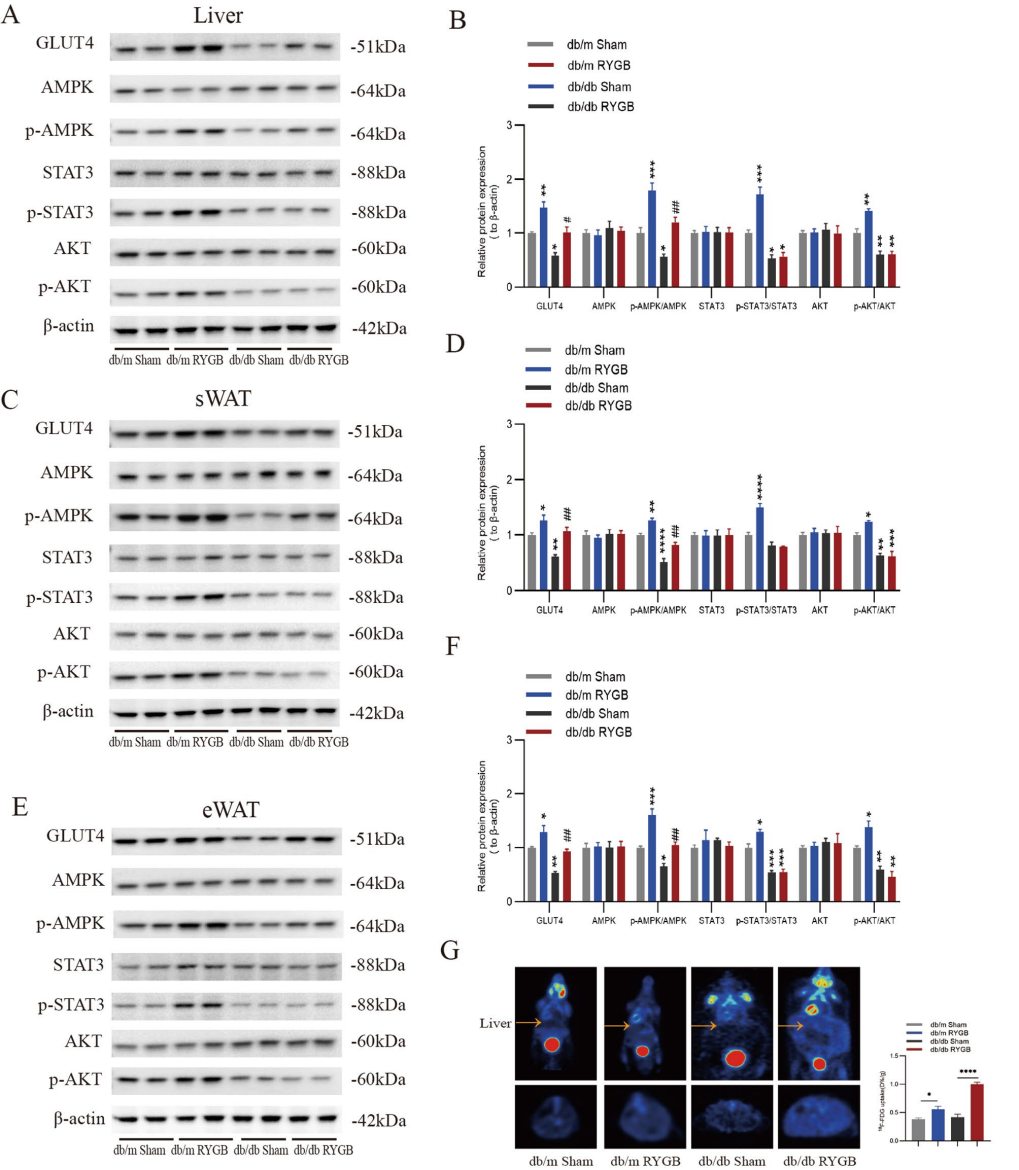
RYGB actives AMPK to upregulate GLUT4 in db/db mice. **A** and **B**, Representative western blots and quantitative results of GLUT4, AMPK, pAMPK, STAT3, pSTAT3, pAKT and AKT in the liver from db/m mice and db/db mice after sham and RYGB surgery (*n* = 4). Protein levels were normalized to those of β-actin. **C** and **D**, Representative western blots and quantitative results of GLUT4, AMPK, pAMPK, STAT3, pSTAT3, pAKT and AKT in the sWAT from db/m mice and db/db mice after sham and RYGB surgery (*n* = 4). Protein levels were normalized to those of β-actin. sWAT, subcutaneous white adipose tissue. **E** and **F**, Representative western blots and quantitative results of GLUT4, AMPK, pAMPK, STAT3, pSTAT3, pAKT and AKT in the eWAT from db/m mice and db/db mice after sham and RYGB surgery (*n* = 4). Protein levels were normalized to those of β-actin. eWAT, epididymal white adipose tissue. **G**, Representative PET/CT images (left) and the relative quantification of [18 F]-FDG uptake (right) from db/m mice and db/db mice after sham and RYGB surgery (*n* = 6). In the PET/CT image, the yellow arrow points towards the liver, with its cross-section at the bottom. The data are presented as the mean ± SEM. *P < 0.05, **P < 0.01***P < 0.001, ****P < 0.0001, compared with the db/m sham group; #*P* < 0.05, ##*P* < 0.01, ###*P* < 0.001, ####*P* < 0.001, compared with the db/db sham group, by one-way ANOVA

**Fig. 6:**
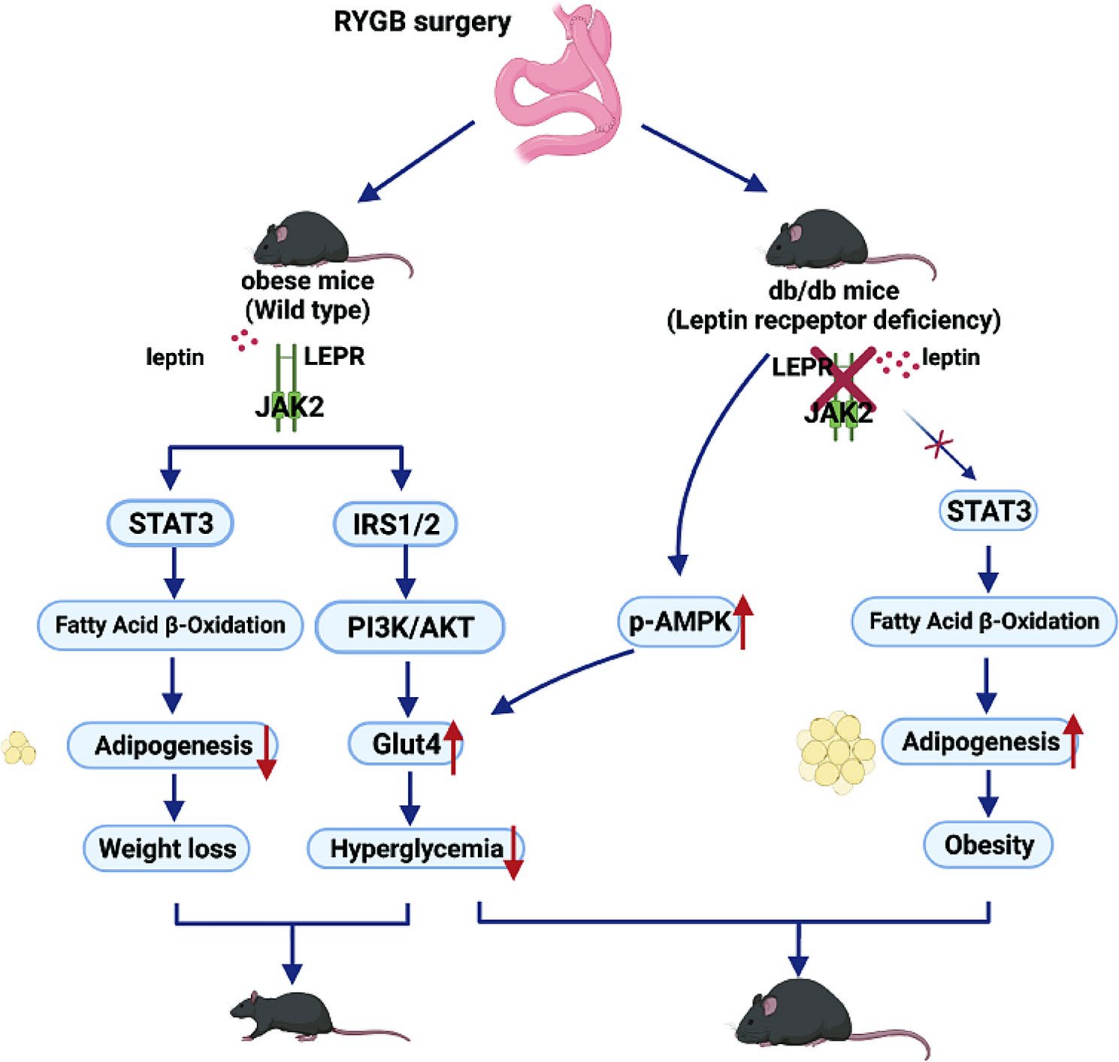
Schematic of the mechanism of this work. When the leptin pathway is intact, binding of leptin to LEPR activates JAK2-STAT3 to promote FAO or activate IRS1/2-PI3K/AKT to mediate GLUT4 translocation on cell membranes to regulating glucose uptake through its receptor. However, the above pathways will be damaged under leptin receptor deficiency, which leads to obesity and diabetes. RYGB surgery activates AMPK and upregulates the cell membrane translocation of GLUT4, thus promoting cellular glucose uptake, while AMPK cannot trans-autophosphorylate STAT3 through the tyrosine site on LEPR in db/db mice through JAK2 due to the lack of leptin receptor. RYGB: Roux-en-Y gastric bypass; LEPR: leptin receptor; GLUT4: glucose transporter 4

Leptin exerts its physiological action through the leptin receptor (LEPR). LEPR can activate JAK2-STAT3 to promote FAO or activate IRS1/2-PI3K/AKT to stimulate glucose transporter 4 (GLUT4) translocation to the cell membrane [25–28]. We showed that p-AMPK and Glut4 were significantly upregulated after RYGB in the liver (Fig. 5A, B). The p-STAT3 and p-AKT were significantly enhanced after RYGB in db/m mice. However, p-STAT3 and p-AKT were not changed after RYGB in db/db mice (Fig. 5A, B). Similar results were also observed in adipose tissue (Fig. 5C-F). Furthermore, glucose uptake in the liver was markedly improved in RYGB-treated mice, especially db/db mice (Fig. 5G). These results suggest that RYGB can increase AMPK phosphorylation to promote the upregulation of GLUT4, thereby enhancing glucose uptake (Fig. 6). However, activation of AMPK could not increase FAO through the STAT3 pathway under leptin receptor deficiency (Fig. 6).

## Discussion

Our study provides the new evidence to demonstrate that RYGB surgery has a hypoglycemic effect but fails to promote weight loss under leptin receptor deficiency in genetically obese diabetic mice. Mechanistically, RYGB surgery activates AMPK and upregulates GLUT4, thus promoting tissue glucose uptake and metabolism, while AMPK cannot promote fatty acid oxidation through the STAT3 pathway under leptin receptor deficiency.

Currently, metabolic surgery is the most effective intervention for the treatment of obesity and T2DM. Some diabetic patients appear regaining weight after metabolic surgery, but their blood glucose levels are still well controlled [29]. Interestingly, the hypoglycemic effect presents ahead of dominant weight loss in obese patients with diabetes after metabolic surgery [30]. These studies demonstrate that the beneficial effect of metabolic surgery on hyperglycemia is partly weight-independent. In this study, we showed that db/db mice exhibited more subcutaneous and visceral lipid accumulation after RYGB, but the levels of blood glucose and insulin remained normal. In addition, we further demonstrated the separation of diabetes and obesity after metabolic surgery through leptin receptor deficiency.

Leptin receptor deficiency is a rare endocrine disease [31]. Clinical trials have shown that obese patients with leptin receptor mutations have poor weight loss and easy weight regain after metabolic surgery [20–22]. In this study, we also observed weight regain after RYGB in db/db mice. However, the weight loss effect of RYGB surgery is significant in diet-induced obese mice with leptin receptors [32, 33]. It is widely accepted that the leptin receptor is a key molecule in energy metabolism [34]. When the leptin pathway is intact, JAK2 activates the STAT3 pathway by phosphorylating tyrosine at position 1138 of the leptin receptor, while downstream STAT3 promotes the upregulation of CTP1A expression, and then enhances fatty acid oxidation [35, 36]. In addition, the leptin receptor downstream of PI3K-AKT mediates GLUT4 translocation to cell membranes to regulate glucose uptake [37]. We have confirmed that metabolic surgery can activate AMPK to regulate glucose homeostasis in an insulin-independent manner [12]. The activation of AMPK can upregulate the membrane translocation of GLUT4 by phosphorylating TBC1D1 (TBC domain family, member 1), which is independent of the PI3K-AKT pathway to promote cellular glucose uptake [17]. Also, we found that p-AMPK/AMPK and GLUT4 upregulated under leptin receptor deficiency after RYGB, while the AMPK-mediated FAO disappeared due to the blockade of the JAK2-STAT3 pathway and impeded fatty acid metabolism. This evidence suggests that the integrity of the leptin receptor pathway plays a key role in weight loss under AMPK activation after metabolic surgery.

In conclusion, this study demonstrated that the hypoglycemic effect of metabolic surgery was independent of weight loss. Its underlying mechanism is associated with the disturbance of the leptin receptor-related JAK2/STAT3 pathway. This study sheds new light on understanding metabolic surgery for the treatment of obesity and diabetes.

### Electronic supplementary material

Below is the link to the electronic supplementary material.

Supplementary Material 1

Supplementary Material 2

## Data Availability

No datasets were generated or analysed during the current study.

## Abbreviations

RYGB: Roux-en-Y gastric bypass
T2DM: Type 2 diabetes
GLUT4: Glucose transporter 4
FAO: Fatty acid oxidation
HE: Hematoxylin-eosin
TC: Total cholesterol
TG: Triglycerides
HDL-C: High-density lipoprotein cholesterol
LDL-C: Low-density lipoprotein cholesterol
RER: Respiratory Exchange Ratio
EE: Energy Expenditure
IPGTT: Intraperitoneal glucose tolerance test
OGTT: Oral glucose tolerance test
ITT: Insulin tolerance test
BAT: Brown adipose tissue
iWAT: Inguinal white adipose tissue
PAT: Perirenal adipose tissue
mWAT: Mesenteric white adipose tissue
eWAT: Epididymis white adipose tissue
GCK: Glucokinase
PFKL: Phosphofructokinase liver type
PKLR: Pyruvate kinase isozymes R/L
ACLY: ATP-citrate lyase
CPT-1: Carnitine palmitoyltransferase-1
FASN: Fatty acid synthesis
SREBP1: Sterol regulatory element-binding protein 1
LEPR: Leptin receptor
TBC1D1: TBC domain family, member 1
