# Supplementary figures and images for: Leptin receptor deficiency impedes metabolic surgery related-weight loss through inhibition of energy expenditure in db/db mice
Source: Diabetol Metab Syndr. 2024 Feb 1;16:33. doi: 10.1186/s13098-024-01270-7 (PMC10832203; doi:10.1186/s13098-024-01270-7)

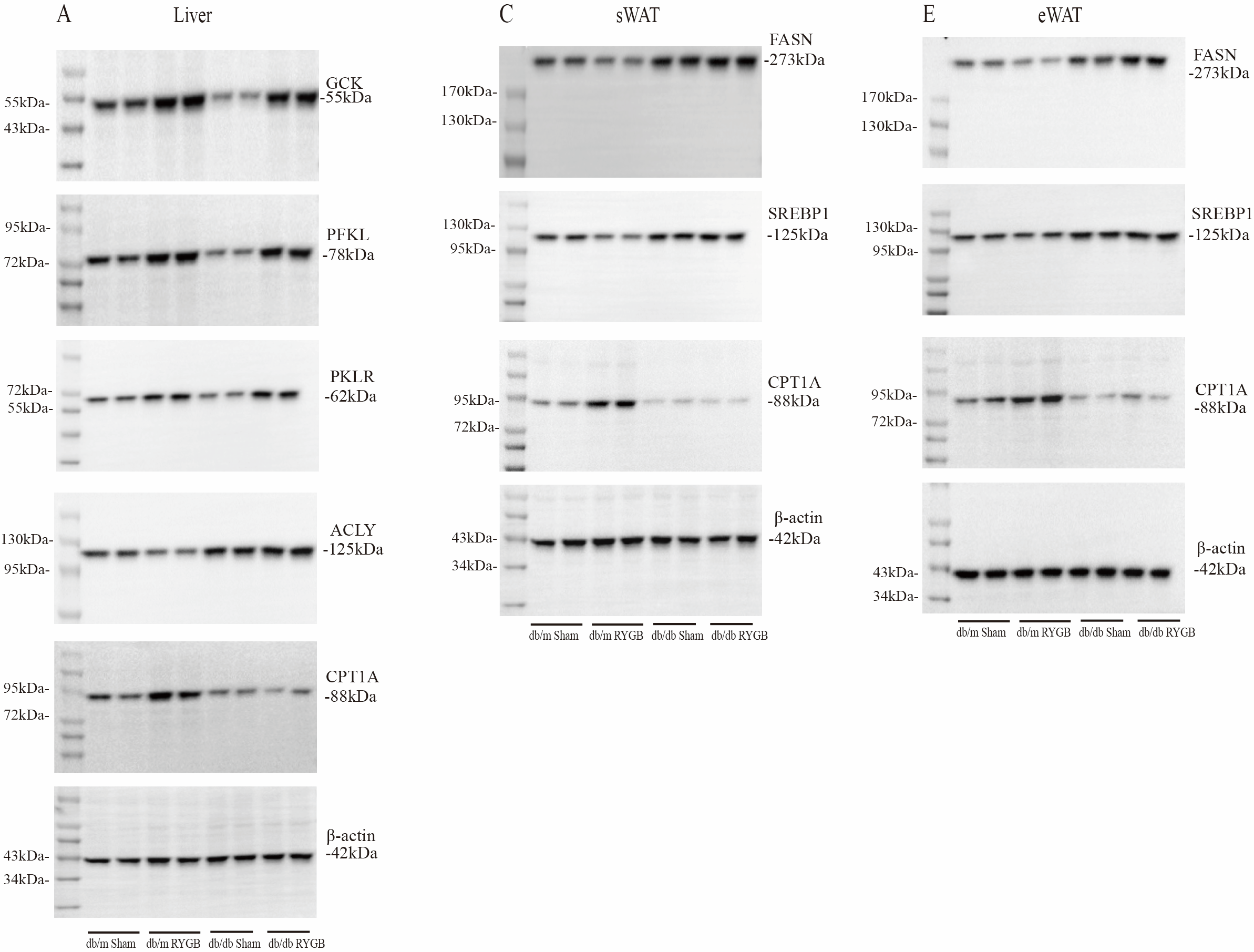

Supplement: Supplementary file 1 — Supplementary Material 1 [file 13098_2024_1270_MOESM1_ESM.png]

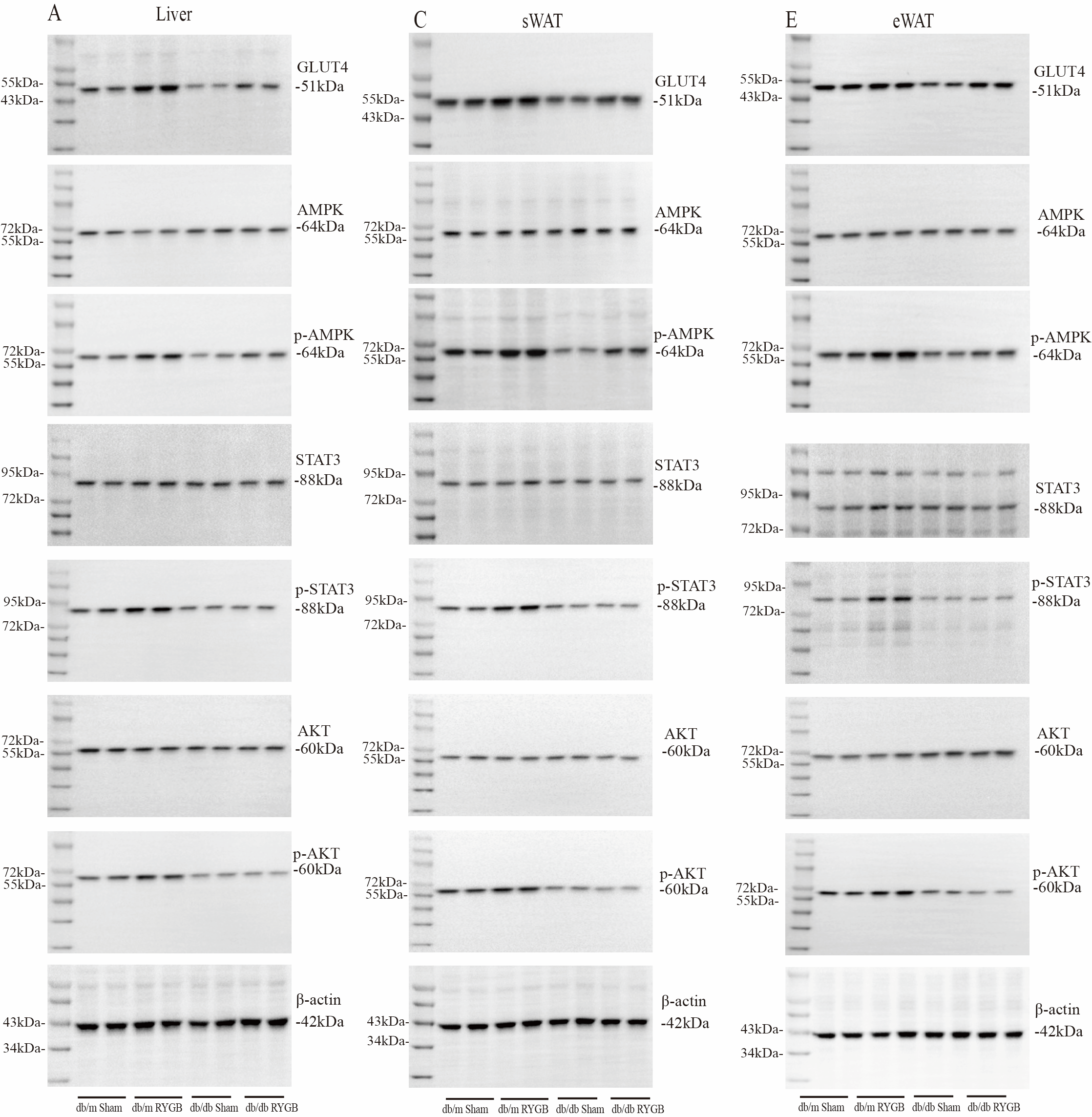

Supplement: Supplementary file 2 — Supplementary Material 2 [file 13098_2024_1270_MOESM2_ESM.png]
